# Electrostatic-Assembly-Driven Formation of Supramolecular Rhombus Microparticles and Their Application for Fluorescent Nucleic Acid Detection

**DOI:** 10.1371/journal.pone.0018958

**Published:** 2011-04-19

**Authors:** Hailong Li, Junfeng Zhai, Xuping Sun

**Affiliations:** 1 State Key Lab of Electroanalytical Chemistry, Changchun Institute of Applied Chemistry, Chinese Academy of Sciences, Changchun, Jilin, China; 2 Graduate School of the Chinese Academy of Sciences, Beijing, China; The University of Manchester, United Kingdom

## Abstract

In this paper, we report on the large-scale formation of supramolecular rhombus microparticles (SRMs) driven by electrostatic assembly, carried out by direct mixing of an aqueous HAuCl_4_ solution and an ethanol solution of 4,4′-bipyridine at room temperature. We further demonstrate their use as an effective fluorescent sensing platform for nucleic acid detection with a high selectivity down to single-base mismatch. The general concept used in this approach is based on adsorption of the fluorescently labeled single-stranded DNA (ssDNA) probe by SRM, which is accompanied by substantial fluorescence quenching. In the following assay, specific hybridization with its target to form double-stranded DNA (dsDNA) results in desorption of ssDNA from SRM surface and subsequent fluorescence recovery.

## Introduction

The development of rapid, cost-effective, sensitive and specific methods for nucleic acid detection is becoming more and more important, owing to their potential diverse applications in gene expression profiling, clinical disease diagnostics and treatment [1]. With the increasing availability of nanostructures, widespread attention has been paid to their diagnostic potential in biotechnological system [2], and the employment of various nanostructures for this purpose has been well documented [3]. Recently, much effort has been made to develop homogeneous fluorescence assays based on FRET (fluorescence resonance energy transfer) or quenching mechanism for nucleic acid detection [4]. The selection issue of a fluorophore-quencher pair is eliminated from the nanostructure-involved fluorescence assay system because the same nanostructure serving as a nanoquencher can quench dyes of different emission frequencies [4], [5]. Up to now, however, only limited nanostructures have been successfully used as quencher for this assay [4]–[22]. Dubertret et al. have pioneered the use of dye fluorescence quenching ability of small gold nanoparticles (AuNPs) for DNA detection [6]. In their study, a DNA moiety is decorated to a 1.4-nm AuNP surface and its stem region is curved to a hairpin structure by Watson-Crick hydrogen bonding. This conformational change brings fluorescent dye into close proximity of the nanoparticle, leading to quenching of dye fluorescence. The subsequent specific hybridization of the moiety with target opens the hairpin and thus separates the fluorophore from the AuNP at a sufficient distance to allow fluorescence recovery. Maxwell et al. have also developed a similar AuNP-based nanobiosensor to detect nucleic acid [7]. Although both of them are able to differentiate single-base mismatch in target sequence, they require tedious and laborious surface attachment chemistry for probe immobilization and suffer from slow response. To solve these problems, Li et al. have designed a novel fluorescent assay for DNA hybridization, which is based on that single-stranded DNA (ssDNA) adsorbs on negatively charged AuNP while double-stranded DNA (dsDNA) does not. As a result, dye-labeled probe sequences have their fluorescence efficiently quenched when they are mixed with AuNPs unless they hybridize with components of the analyte [8]. Application of gold nanoparticle as a fluorescence quencher was further explored recently [9], [10]. Other structures have also been successfully used in this assay, including single-walled [5], [11] and multi-walled [12] carbon nanotubes, graphene oxide [13], [14], carbon nanoparticles [15], carbon nanospheres [16], nano-C_60_ [17], mesoporous carbon microparticles [18], polyaniline nanofibres [19], poly(*o*-phenylenediamine) colloids [20], coordination polymer colloids [21], Ag@poly(m-phenylenediamine) core-shell nanoparticles [22], tetracyanoquinodimethane nanoparticles [23], and poly(*p*-phenylenediamine) nanobelts [24].

Self-assembly refers to the spontaneous organization of molecules, molecular clusters, and aggregate structures into two-dimensional (2D) arrays and three-dimensional (3D) networks by attractive forces or chemical bond formation. It provides an effective and versatile approach for constructing a structured system at a molecular level [25]. Among them, the most often studied involves self-assembled monolayers formed on planar solid substrates [26], monolayer-protected clusters [27], self-assembly into 3D networks on planar solid substrates [28], layer-by-layer self-assembly of ultrathin films on planar solid substrates [29] or colloidal particles [30], etc. On the other hand, solution-based self-assembly has drawn increasing attention because it provides a means for the integration of molecular systems into functional mesoscopic devices and macroscopic materials [31].

In this paper, we report the formation of supramolecular rhombus microparticles (SRMs) via a solution-based self-assembly strategy, carried out by direct mixing an aqueous HAuCl_4_ solution and an ethanol 4,4′-bipyridine solution at room temperature. We further demonstrate the proof of concept of using such SRMs as an effective fluorescent sensing platform for nucleic acid detection. In this regard, the nucleic acid detection is accomplished by two steps: Firstly, SRM adsorbs dye-labeled ssDNA, which brings dye and SRM into close proximity and results in fluorescence quenching. Secondly, hybridization of the probe with its complementary target generates a dsDNA which detaches from SRM, leading to fluorescence recovery. Most importantly, the present system has a high selectivity down to single-base mismatch.

## Results and Discussion

**Figure 1:**
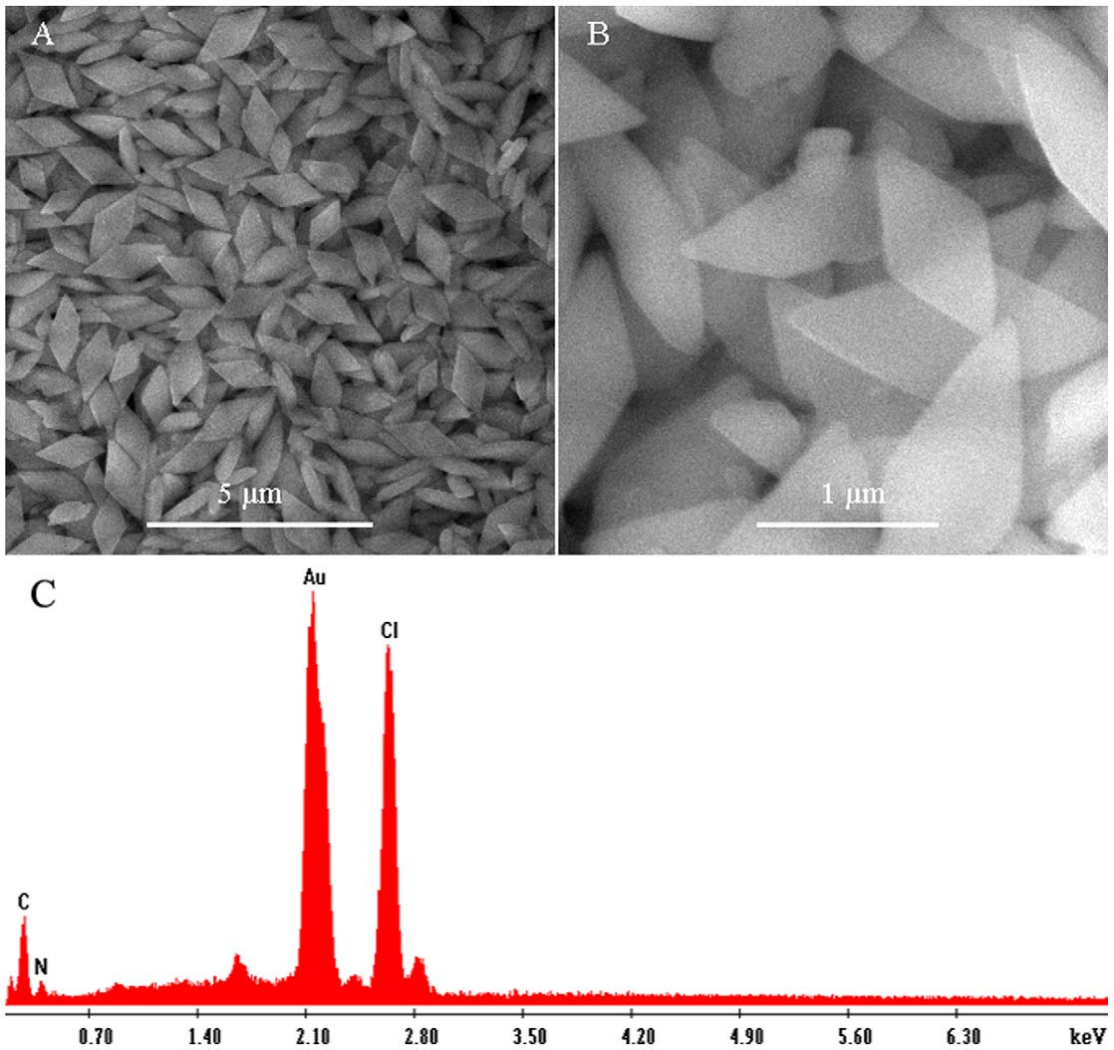
Instrumental analysis of the precipitate thus formed. (A) Low and (B) high magnification SEM images and (C) the corresponding EDS spectrum of the resultant product.

Figure 1A and Figure 1B show typical SEM images and of the precipitate thus formed. The low magnification SEM image shown in Figure 1A indicates that the precipitate consists exclusively of a large amount of particles. The high magnification SEM image further reveals that they are rhombus microparticles with a side length in the range of 500–900 nm and smooth surface, as shown in Figure 1B. Some small irregular particles are also observed as the by-products. The chemical composition of the resultant microparticles was determined by energy-dispersed spectrum (EDS, Figure 1C). The EDS spectrum shows peaks corresponding to C, N, Cl, and Au elements (other peaks originated from the substrate). Based on these observations, we can conclude that these structures are products formed from HAuCl_4_ and 4,4′-bipyridine. HAuCl_4_ is a kind of acid, while 4,4′-bipyridine belongs to organic base. When 4,4′-bipyridine is mixed with HAuCl_4_, protonated 4,4′-bipyridine is formed. Taking the negative charge of AuCl_4_^−^ and the positive charge of protonated 4,4′-bipyridine into consideration, we may suggest that electrostatic attractions between these two components drive them to assemble into supramolecular microparticles [32], [33]. We have carried out a controlled experiment by mixing these two components under basic conditions (pH: 10), however, only a clear solution was obtained and no precipitate occurred. This can be ascribed to the failure of protonation of 4,4′-bipyridine under such basic condition and thus no electrostatic assembly occurs.

**Figure 2:**
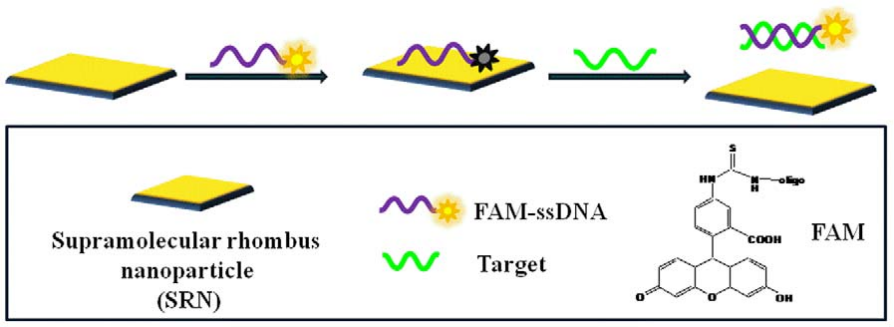
Illustration of the sensing mechanism. A schematic (not to scale) to illustrate the fluorescent nucleic acid detection using SRM as a sensing platform.

SRM is a π-rich structure and thus there should be strong π-π interactions between the DNA bases and SRM [34], which brings them into very close proximity. The zeta potential of SRMs was measured to be –0.63 mV, suggesting that SRM has a low negative surface charge density. So, there should be some degree of electrostatic repulsive interactions between SRM and negatively charged backbone of ssDNA. However, the slight electrostatic repulsion only produces little restriction to the adsorption of ssDNA on SRM in the presence of a large amount of salt in buffer [22]. In contrast, it is expected that SRM might have no binding with dsDNA due to its negatively charged surface and the unavailability of unpaired DNA bases. Figure 2 shows a schematic to illustrate our original idea about the SRM-based fluorescence-enhanced nucleic acid detection. The detection of DNA can be accomplished by two steps: (1) SRM binds FAM-ssDNA probe via π-π interactions between DNA bases and SRM, their close proximity may result in quenching of the fluorescence of ssDNA probe. (2) The hybridization of FAM-ssDNA with its target produces a dsDNA which detaches from SRM, leading to fluorescence recovery.

**Figure 3:**
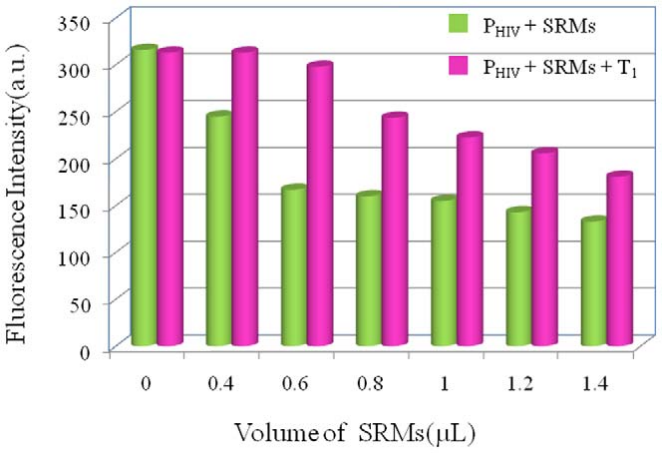
Investigation of the influence of the amount of SRMs on the system. Fluorescence intensity histograms of P_HIV_ + SRMs and P_HIV_ + SRMs + T_1_ with the use of 0, 0.4, 0.6, 0.8, 1.0, 1.2, and 1.4-µL SRMs sample in this system, respectively. ([P_HIV_] = 50 nM; [T_1_] = 300 nM). Excitation was at 480 nm and the fluorescence emission intensity was monitored at 518 nm. All measurements were done in Tris-HCl buffer in the presence of 15 mM Mg^2+^ (pH: 7.4).

We demonstrate the application of such SRMs as a fluorescent sensing platform for nucleic acid detection using an oligonucleotide sequence associated with human immunodeficiency virus (HIV) as a model system. This sequence is labeled with a fluorophore (FAM) to constitute the probe P_HIV_. Adsorption of P_HIV_ on SRMs will leads to substantial fluorescence quenching, however, a significant fluorescence enhancement can be observed in the presence of complementary target T_1_. The amount of SRMs used in this system should have great impact on the suggested method. The influence of the amount of SRMs on the fluorescence quenching and the subsequent recovery was firstly taken into investigation. Figure 3 shows the fluorescence intensity histograms of seven samples measured in the presence of 0, 0.4, 0.6, 0.8, 1.0, 1.2, and 1.4-µL SRMs sample, respectively, demonstrating that the increased amount of SRMs leads to an increased quenching efficiency but a decreased recovery efficiency. Such observation can be explained as follows: Involvement of more SRMs leads to more efficient adsorption of ssDNA on them and thus increases the fluorescence quenching. On the other hand, there should be more unoccupied space available on SRM, leading to direct adsorption of more target molecules. Thus, the immobilized targets fail to form dsDNA with probe during the hybridization process, resulting in decreased hybridization and recovery efficiency. Based on the above experimental results, 0.6-µL SRMs sample was chosen as the optimal amount in our present study for all measurements.

**Figure 4:**
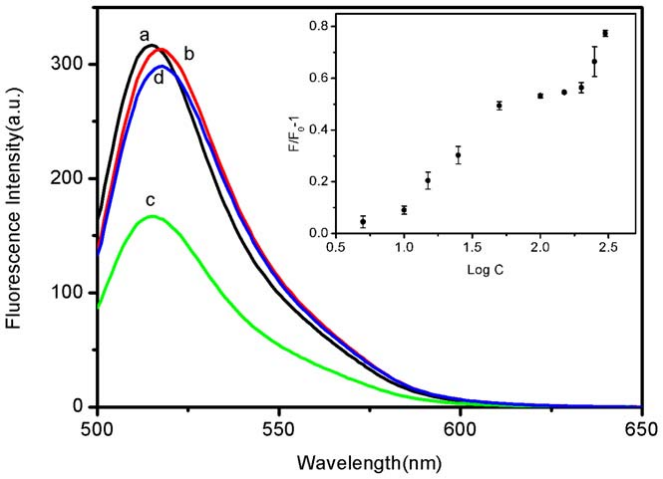
Target ssDNA detection. Fluorescence emission spectra of P_HIV_ (50 nM) under different conditions: (a) P_HIV_; (b) P_HIV_ + 300 nM T_1_; (c) P_HIV_ + 0.6-µL SRMs; (d) P_HIV_ + 0.6-µL SRMs + 300 nM T_1_. Inset: fluorescence intensity changes (F/F_0_–1) of P_HIV_–SRM complex plotted against logarithm of T_1_ concentration (F_0_ and F are the fluorescence intensity without and with the presence of T_1_, respectively). Excitation was at 480 nm and the fluorescence emission intensity was monitored at 518 nm. All measurements were done in Tris-HCl buffer in the presence of 15 mM Mg^2+^ (pH: 7.4).

Figure 4 shows the fluorescence emission spectra of this FAM-labeled ssDNA probe, P_HIV_, under different conditions. In the absence of SRMs, P_HIV_ exhibits strong fluorescence emission due to the presence of the fluorescein-based dye (curve a). However, the presence of 0.6-µL SRMs results in about 47% quenching of the fluorescence emission (curve c), indicating that SRMs can adsorb ssDNA and quench the fluorescent dye very effectively. The strong π-π interactions between the DNA bases and SRM bring FAM into close proximity of SRM. It was found that the fluorescence quenching was suppressed by introducing N,N-dimethylformamide (DMF) (Figure S1). The introduction of DMF changes the solvent polarity in the assay system, weakening this π-π interaction. Thus, the adsorption of ssDNA on SRMs is decreased and the resultant fluorescence quenching is suppressed. The adsorption of P_HIV_ on SRMs can be supported by the experimental fact that no obvious fluorescent change was observed after removal of SRMs from the solution by centrifugation, as shown in Figure S2 (the observed fluorescence is from uncaptured P_HIV_ by SRMs). Note that the absorption spectrum of SRMs dispersed in Tris-HCl buffer (pH 7.4) shown in Figure S3 exhibits absorption peaks at 200 and 250 nm, suggesting that there is no spectra overlap and thus no FRET occurs between SRM and the fluorescent dye FAM. The observed fluorescence quenching in our present study can be attributed to photoinduced electron transfer (PET) from nitrogen atom in SRM to excited fluorophore due to their close proximity [35], [36]. Upon its incubation with complementary target T_1_ for 30 min, the P_HIV_–SRM complex exhibits significant fluorescence enhancement, leading to 94% fluorescence recovery (curve d). The desorption of dsDNA from SRMs can be supported by the experimental fact that the fluorescence intensity of the supernatant of the hybridization mixture remained the same after removal of SRMs by centrifugation (Figure S4). SEM images of SRMs after mixing P_HIV_ and T_1_ at high concentration were also taken, as is shown in Figure S5. It should be pointed out that the fluorescence of the free P_HIV_ was scarcely influenced by the addition of T_1_ in the absence of SRMs (curve b in Figure 4). Figure 4 inset shows the fluorescence intensity changes (F/F_0_–1) of P_HIV_–SRM complex in the presence of varied T_1_ concentrations, where F_0_ and F are FAM fluorescence intensities at 518 nm in the absence and presence of T_1_, respectively. In the DNA concentration range of 5−300 nM, a dramatic increase of FAM fluorescence intensity was observed, demonstrating that the SRM-DNA assembly approach is effective in probing biomolecular interactions.

**Figure 5:**
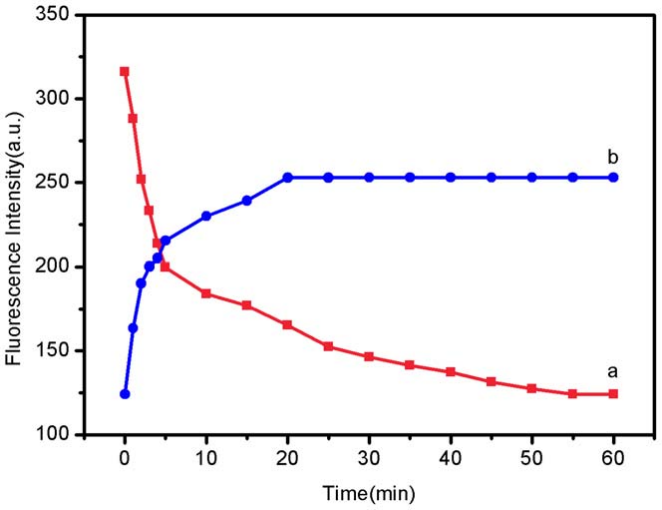
Kinetic behaviour study of fluorescence quenching and recovery. (a) Fluorescence quenching of P_HIV_ (50 nM) by 0.6-µL SRMs and (b) fluorescence recovery of P_HIV_–SRM by T_1_ (300 nM) as a function of incubation time. Excitation was at 480 nm and the fluorescence emission intensity was monitored at 518 nm. All measurements were done in Tris-HCl buffer in the presence of 15 mM Mg^2+^ (pH: 7.4).

We also studied the kinetic behaviors of P_HIV_ with SRM and P_HIV_–SRM complex with T_1_ by collecting the time-dependent fluorescence emission spectra. Plot a in Figure 5 shows the fluorescence quenching of P_HIV_ in the presence of SRM as a function of incubation time. In the absence of the target, the curve exhibits a rapid reduction in the first 5 min and reaches equilibrium within the following 50 min. Plot b in Figure 5 shows the fluorescence recovery of P_HIV_–SRM by T_1_ as a function of time. In the presence of the target T_1_, the curve shows a fast increase in the first 2 min, followed by a slow fluorescence enhancement. The best fluorescence response was obtained after about 20-min incubation.

**Figure 6:**
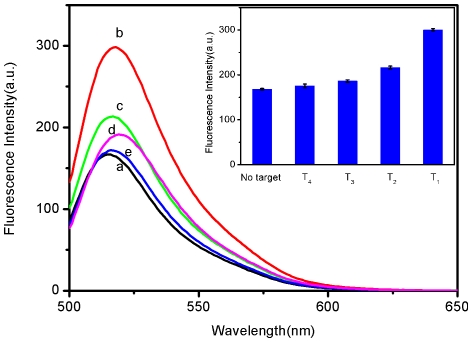
Detection of mismatched sequence. Fluorescence emission spectra of P_HIV_ (50 nM) under different conditions: (a) P_HIV_–SRM complex; (b) P_HIV_–SRM complex + 300 nM T_1_; (c) P_HIV_–SRM complex + 300 nM T_2_; (d) P_HIV_–SRM complex + 300 nM T_3_; (e) P_HIV_–SRM complex + 300 nM T_4_. The amount of SRMs used is 0.6 µL. Inset: fluorescence intensity histograms with error bar. Excitation was at 480 nm and the fluorescence emission intensity was monitored at 518 nm. All measurements were done in Tris-HCl buffer in the presence of 15 mM Mg^2+^ (pH: 7.4).

It is worthwhile mentioning that the sensing platform described herein can well discriminate perfect complementary and mismatched sequences. Figure 6 shows the fluorescence responses of P_HIV_–SRM complex toward complementary target T_1_, single-base mismatched target T_2_, two-base mismatched target T_3_, and non-complementary target T_4_. It is observed that the F/F_0_ value (F_0_ and F are the fluorescence intensities without and with the presence of target, respectively) obtained upon addition of 300 nM T_2_ and T_3_ is about 72% and 60% of the value obtained upon addition of 300 nM T_1_ into P_HIV_–SRM complex, respectively. The addition of T_4_, however, only leads to slight change of fluorescence intensity. Figure 6 inset presents the corresponding fluorescence intensity histograms with error bars. All the above observations indicate that the present nucleic acid detection system has a high selectivity down to single-base mismatch and the results obtained have good reproducibility. Therefore, it is promising for application in single-nucleotide polymorphism detection upon further development.

In summary, for the first time, we demonstrate the electrostatic-assembly-driven formation of SRMs from HAuCl_4_ and 4,4′-bipyridine and their subsequent use as an effective fluorescent sensing platform for nucleic acid detection with a high selectivity down to single-base mismatch. This sensing platform holds great promise for universal and effective fluorescence-enhanced detection with high sensitivity and selectivity to the target molecule studied.

## Materials and Methods

All chemically synthesized oligonucleotides were purchased from Shanghai Sangon Biotechnology Co. Ltd. (Shanghai, China). DNA concentration was estimated by measuring the absorbance at 260 nm. All the other chemicals were purchased from Aladin Ltd. (Shanghai, China) and used as received without further purification. The water used throughout all experiments was purified through a Millipore system. SRMs were prepared as follows: In brief, 4 mL of 24.3 mM HAuCl_4_ aqueous solution was added into 8 mL of 0.1 M 4,4′-bipyridine in ethanol under vigorous stirring, resulting in the formation of a large amount of yellow precipitate immediately. The precipitate thus formed was washed with water several times and then redispersed in 8-mL water for characterization and further use. The volume of each sample for fluorescence measurement is 400 µL in 20 mM Tris-HCl buffer containing 100 mM NaCl, 5 mM KCl, and 15 mM MgCl_2_ (pH: 7.4) if not specified. All the experiments were carried out at room temperature (about 25 °C).

For characterization by scanning electron microscopy (SEM), 2 µL of the suspension was placed on an indium tin oxide (ITO) glass slide and air-dried at room temperature. SEM measurements were made on a XL30 ESEM FEG scanning electron microscope at an accelerating voltage of 20 kV. An energy-dispersive X-ray spectroscopic detecting unit was used to collect the energy-dispersed spectrum (EDS) for elemental analysis. Fluorescent emission spectra were recorded on a RF-5301PC spectrofluorometer (Shimadzu, Japan). Zeta potential measurements were performed on a Nano-ZS Zetasizer ZEN3600 (Malvern Instruments Ltd., U.K.).

Oligonucleotide sequences are listed as follows (mismatch underlined).

P_HIV_ (FAM dye-labeled ssDNA):

5′-FAM-AGT CAG TGT GGA AAA TCT CTA GC-3′

T_1_ (complementary target):

5′-GCT AGA GAT TTT CCA CAC TGA CT-3′

T_2_ (single-base mismatched target):

5′-GCT AGA GAT TGT CCA CAC TGA CT-3′

T_3_ (two-base mismatched target):

5′-GCT AGA GAT TGT ACA CAC TGA CT-3′

T_4_ (non-complementary target to P_HIV_)_:_

5′-TTT TTT TTT TTT TTT TTT TTT TT-3′

## Supporting Information

Figure S1 **Evaluation of π-π interaction between P_HIV_ and SRMs.** The histograms of F/F_0_ with error bars in Tris-HCl buffer and in Tris-HCl + DMF (50%) buffer, where F_0_ and F are the fluorescence intensities of P_HIV_ (50 nM) in the absence and presence of 0.6-µL SRMs, respectively. Excitation was at 480 nm and the fluorescence emission intensity was monitored at 518 nm.
(TIF)

Figure S2 **Adsorption of P_HIV_ on SRMs confirmation.** Fluorescence spectra of (a) P_HIV_–SRM and (b) the supernatant of (a) after removing SRMs by centrifugation. ([P_HIV_]: 50 nM, the volume of SRMs used is 0.6 µL). Excitation was at 480 nm. All measurements were done in Tris-HCl buffer in the presence of 15 mM Mg^2+^ (pH: 7.4).
(TIF)

Figure S3 **UV-vis absorption of SRMs.** Absorption spectrum of SRMs dispersed in Tris-HCl buffer in the presence of 15 mM Mg^2+^ (pH 7.4).
(TIF)

Figure S4 **Confirmation of desorption of P_HIV_ from SRMs upon hybridization.** Fluorescence spectra of (a) P_HIV_–SRM + 300 nM T_1_ and (b) the supernatant of (a) after removing SRMs by centrifugation. ([P_HIV_]: 50 nM, the volume of SRMs used is 0.6 µL). Excitation was at 480 nm. All measurements were done in Tris-HCl buffer in the presence of 15 mM Mg^2+^ (pH: 7.4).
(TIF)

Figure S5 **SEM images of SRMs used in hybridization.** (A) Low and (B) high magnification SEM images of SRMs, collected by centrifugation of P_HIV_ + SRMs + T_1_.
(TIF)
