# Supplementary figures and images for: Electrostatic-Assembly-Driven Formation of Supramolecular Rhombus Microparticles and Their Application for Fluorescent Nucleic Acid Detection
Source: PLoS One. 2011 Apr 19;6(4):e18958. doi: 10.1371/journal.pone.0018958 (PMC3079751; doi:10.1371/journal.pone.0018958)

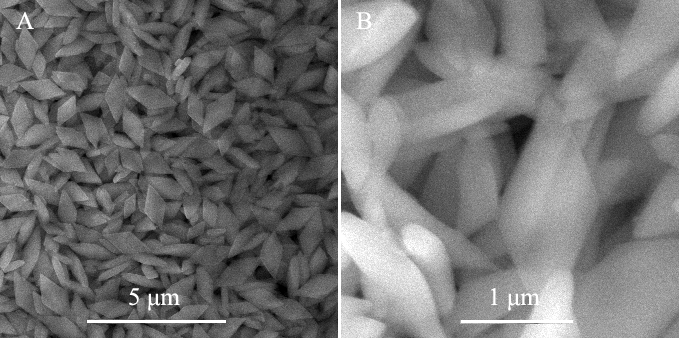

Supplement: Figure S5 — SEM images of SRMs used in hybridization. (A) Low and (B) high magnification SEM images of SRMs, collected by centrifugation of PHIV + SRMs + T1. (TIF) [file pone.0018958.s005.tif]
